# Imaging mass spectrometry for the precise design of antibody-drug conjugates

**DOI:** 10.1038/srep24954

**Published:** 2016-04-21

**Authors:** Yuki Fujiwara, Masaru Furuta, Shino Manabe, Yoshikatsu Koga, Masahiro Yasunaga, Yasuhiro Matsumura

**Affiliations:** 1 Division of Developmental Therapeutics, Exploratory Oncology Research and Clinical Trial Center, National Cancer Center, Kashiwa, Chiba, 277-8577, Japan; 2 Department of Integrated Biosciences, Graduate School of Frontier Sciences, The University of Tokyo, Kashiwa, Chiba, 277-8561, Japan; 3 Analytical & Measuring Instruments Division Shimadzu Corporation, Kyoto, 604-8511, Japan; 4 Synthetic Cellular Chemistry Laboratory, RIKEN, Wako, Saitama, 351-0198, Japan

## Abstract

Antibody-drug conjugates (ADCs) are a class of immunotherapeutic agents that enable the delivery of cytotoxic drugs to target malignant cells. Because various cancers and tumour vascular endothelia strongly express anti-human tissue factor (TF), we prepared ADCs consisting of a TF-specific monoclonal antibody (mAb) linked to the anticancer agent (ACA) monomethyl auristatin E (MMAE) via a valine-citrulline (Val-Cit) linker (human TF ADC). Identifying the most efficient drug design in advance is difficult because ADCs have complicated structures. The best method of assessing ADCs is to examine their selectivity and efficiency in releasing and distributing the ACA within tumour tissue. Matrix-assisted laser desorption/ionization imaging mass spectrometry (MALDI-IMS) can be used to directly detect the distributions of native molecules within tumour tissues. Here, MALDI-IMS enabled the identification of the intratumour distribution of MMAE released from the ADC. In conclusion, MALDI-IMS is a useful tool to assess ADCs and facilitate the optimization of ADC design.

Antibody-drug conjugates (ADCs) are a class of immunotherapeutic agents that enable the delivery of cytotoxic drugs to target malignant cells. ADCs passively accumulate in solid tumour tissue through the enhanced permeability and retention effect^1^ and actively accumulate in target malignant cells because of the specific antibody-antigen binding^2^. The selection of a suitable monoclonal antibody (mAb), an anticancer agent (ACA), and a linker have supported the clinical success of ADCs^3^. The ADC strategy should be confined to highly toxic ACAs and not applied to ordinary ACAs, such as taxane, adriamycin, and others, because fewer than four ACA molecules should be conjugated to the mAb to prevent a decrease in the affinity of the mAb when too many ACA molecules are attached^4^. Monomethyl auristatin E (MMAE) is one of the most useful and potent ACAs for the clinical development of novel ADCs^5^,^6^. MMAE inhibits cell division by blocking the polymerization of tubulin.

In our laboratory, we have created an anti-human tissue factor (TF) mAb attached to valine-citrulline (Val-Cit)-MMAE (human TF ADC) and have reported its antitumour effect against xenografts of a human pancreatic cancer cell line, BxPC-3^7^. The Val-Cit-MMAE has been designed for maximum serum stability and efficient release into the tumour environment^8^. Once human TF ADC binds to the target malignant cells, it is internalized by endocytosis, and MMAE is theoretically released into the tumour environment through the action of the lysosomal enzymes on the linker. TF is a transmembrane glycoprotein involved in the initiation of the extrinsic pathway of blood coagulation^9^, is expressed in various types of cancer, and plays a role in cancer progression, angiogenesis, tumour growth, and metastasis^10^. Because pancreatic cancer tissue expresses high levels of TF, it is a useful target antigen for this condition^11^,^12^,^13^.

To optimize the efficacy of an ADC against TF-positive solid tumours, a preclinical pharmacological evaluation of the ADC should be performed to determine whether the human TF ADC has been optimally designed. Regarding antitumour effects, ACAs must penetrate the tumour tissue efficiently and be retained there at a high and biologically active concentration^14^,^15^. For such analyses, high-performance liquid chromatography (HPLC) or liquid chromatography mass spectrometry (LC-MS) is generally used. However, these techniques do not provide information about the drug distribution in a specific target area, although they allow optimization of the drug design to a certain extent, enabling more efficiently targeted delivery. Autoradiography can be used to examine the tissue distribution of radiolabelled small molecules^16^. However, this method cannot distinguish between a radiolabelled drug conjugated to an ADC and free radiolabelled drug released from the ADCs^17^.

There are two types of matrix-assisted laser desorption/ionization imaging mass spectrometry (MALDI-IMS): One type can detect larger molecules^18^, although it is currently difficult to directly ionize high-molecular weight proteins, such as mAbs. The other type, which was used in our study, is specifically designed for low-molecular weight substances, such as ACAs. Although it is difficult to determine the distribution of ADCs from a technical standpoint, MALDI-IMS is a useful analytical tool for verifying whether the ADCs release their cytotoxic agent within the tumour tissues as designed. Mass spectrometry (MS) and tandem MS (MS/MS) do not require labelling reagents, and MALDI-IMS can provide accurate maps of the target molecules in tissue specimens directly^19^.

In this study, we investigated the efficiency of MMAE release from human TF ADCs within tumour tissue and the spatial distribution of the released MMAE therein by using MALDI-IMS. The imaging data were acquired using a mass microscope capable of analysing low-molecular weight compounds. The MMAE was imaged with accurate mass at a pixel size between 10 and 20 μm.

## Results

### Visualization of MMAE based on the MMAE-specific MS/MS fragment *m/z* 496.3 using MALDI-IMS

**Figure 1:**
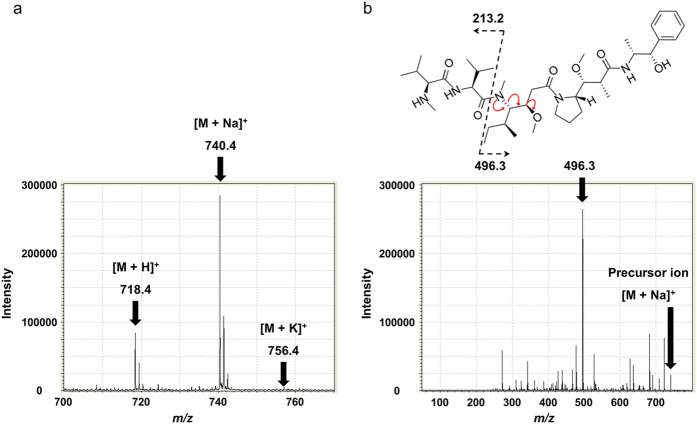
MS and MS/MS of MMAE. (**a**) To define the MS and MS/MS analyses of MMAE, the matrix, CHCA in 75% acetonitrile, 0.02% trifluoroacetic acid, 2.0-mM sodium acetate and a 1/1000 dilution of aniline (10 mg/mL, 1.0 μL) was used for the ionization of the MMAE standard (1.0 μM, 1.0 μL). [M + H]^+^, [M + Na]^+^, and [M + K]^+^ were *m/z* 718.4, 740.4, and 756.4, respectively. (**b**) The MMAE-specific MS/MS fragment, *m/z* 496.3, was determined when [M + Na]^+^ was used as a precursor ion in the MS/MS analysis.

For the application of MALDI to MMAE analysis, α-cyano-4-hydroxycinnamic acid (CHCA) in 75% acetonitrile, 0.02% trifluoroacetic acid, 2.0-mM sodium acetate and a 1/1000 dilution of aniline were used for crystallization. The chemical formula of MMAE is C_39_H_67_N_5_O_7_, and its monoisotopic mass is 717.504. Three positive-ion peaks derived from MMAE were observed by MS analysis: single-charge hydrogen, and the sodium and potassium adducts, denoted as [M + H]^+^, [M + Na]^+^, and [M + K]^+^, respectively (Fig. 1a). The main fragment at *m/z* 496.3 was observed when *m/z* 740.4 was used as a precursor ion in the MS/MS analysis (Fig. 1b). The validation of the MMAE distribution in each sample was performed in MS/MS mode, and *m/z* 496.3 was selected as an assigned MMAE-specific fragment peak. The results of the MS and MS/MS analyses of MMAE were summarized in Table 1.

### Semi-quantitative analysis of MMAE using MALDI-IMS

**Figure 2:**
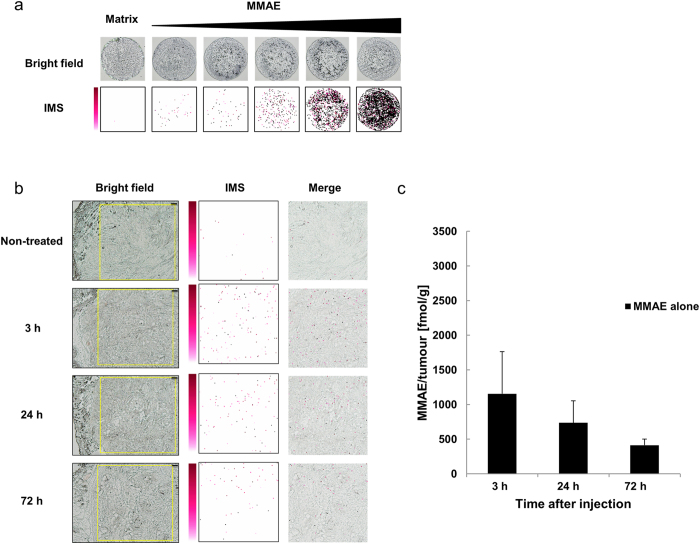
Semi-quantitative analysis of MMAE using MALDI-IMS. (**a**) Only CHCA was used as a negative control. From left to right, the concentrations of MMAE were 0.10, 1.0, 10, 100, and 1000 nM. The CHCA solution (10 mg/mL, 1.0 μL) was used for the ionization of each MMAE sample (1.0 μL). MMAE was semi-quantitatively detected in a concentration-dependent manner using MALDI-IMS. (**b**) The yellow rectangles on the bright field show the measurement area (2500 × 2500 μm). Pixel size, 20 μm; original magnification, x5; scale bar, 200 μm. The images obtained from *m/z* 496.3 using MALDI-IMS for MMAE detection in tumour tissues are shown as a pseudo-colour image (Red-Purple). The 5.0-mg/mL CHCA solution was applied with a pinpoint spray gun. Minimum intensity, 4; maximum intensity, 10. Each merged image superposed the IMS image on the measurement area image obtained 3, 24, or 72 h after the administration of MMAE alone. (**c**) LC-MS/MS analysis quantified the MMAE concentration in the tumours 3, 24, and 72 h after the administration of MMAE alone. The contents of MMAE in the tumour tissues 3, 24, and 72 h after administration were 1155 ± 609, 736 ± 319, and 413 ± 88 fmol/g (mean ± SD, *N* = 5 for each group), respectively.

MMAE was semi-quantitatively detected at different concentrations with IMS (Fig. 2a). Then, 0.2-mg/kg MMAE alone was injected intravenously into the tail vein of the BxPC-3 xenograft model. The 5.0-mg/mL CHCA solution was applied by pinpoint spray gun to the tissue sections. The signals originating from the MMAE were detected in the tumour tissues at 3, 24, and 72 h after the administration of MMAE alone. The MMAE signal decreased in a time-dependent manner (Fig. 2b). Tissue sections serial to those used for MALDI-IMS were also quantified using LC-MS/MS (Fig. 2c), and the results were consistent with the IMS data from MMAE.

### Selective detection of MMAE alone over MMAE conjugated to mAb using MALDI-IMS

**Figure 3:**
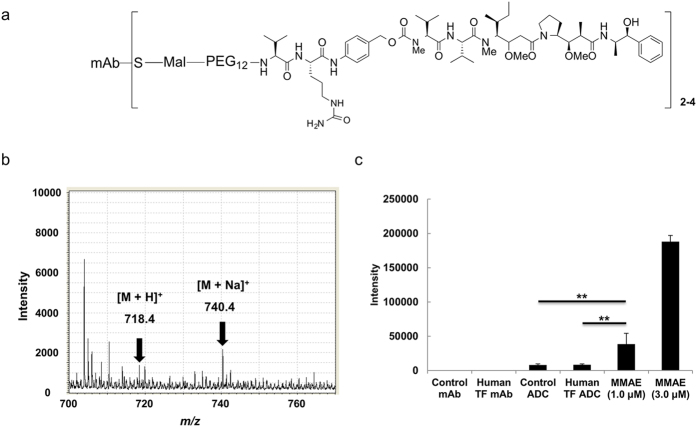
Selective detection of MMAE alone over MMAE conjugated to mAb using MALDI-IMS. (**a**) The DAR of the ADCs was 2–4. A CHCA solution (10 mg/mL, 1.0 μL) was used for the ionization of mAbs (1.0 μM, 1.0 μL), ADCs (1.0 μM, 1.0 μL) and MMAE (1.0 and 3.0 μM, 1.0 μL). (**b**) The mass spectrum of human TF ADC (1.0 μM, 1.0 μL) is indicated. (**c**) The intensities (*m/z* 740.4) of the mAbs, ADCs, and each MMAE sample were measured with MS (Tukey-Kramer, **P < 0.01, *N* = 3 for each group). The intensity was expressed as the mean ± SD. Pixel size, 20 μm; measurement area, 200 × 200 μm.

We prepared the human TF ADC and its control ADC, which did not bind the TF antigen, and the drug-to-antibody ratio (DAR) of the ADCs was 2–4 (Fig. 3a). The ADCs were crystallized with the CHCA solution. MALDI-IMS was performed on the ADC sample and on MMAE alone in MS mode (Fig. 3b,c). The signal intensity of MMAE increased in a concentration-dependent manner, and the signal intensity obtained from 1.0 μL of 1.0-μM MMAE alone was significantly higher than that from 1.0 μL of 1.0-μM human TF ADC and the control ADC (Tukey-Kramer, *P* < 0.01). The MALDI analysis was capable of distinguishing between MMAE alone and ADCs conjugated with MMAE.

### Visualization of MMAE released from ADCs in tumour tissues through MALDI-IMS

**Figure 4:**
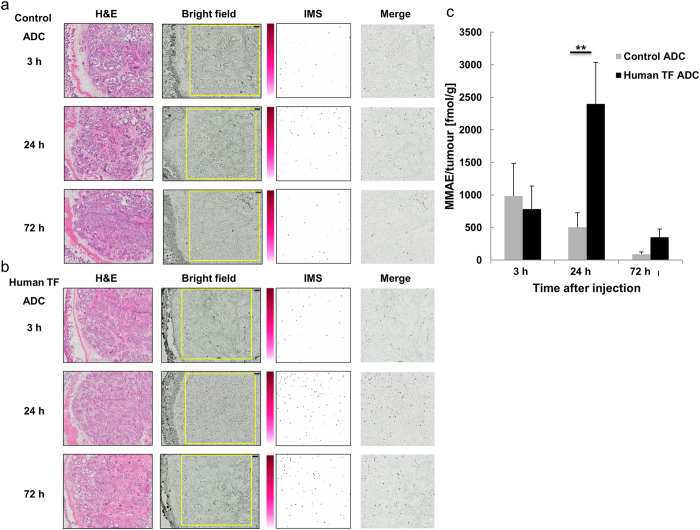
Visualization of MMAE released from ADCs in tumour tissues using MALDI-IMS. (**a**) H&E staining is shown in the left column (original magnification, x5). The yellow rectangles on the bright field show the measurement area (2500 × 2500 μm). Pixel size, 20 μm; scale bar, 200 μm. (**a**,**b**) The images obtained from *m/z* 496.3 using MALDI-IMS for detection of MMAE in tumour tissues are shown as pseudo-colour images (Red-Purple). The 5.0-mg/mL CHCA solution was applied with a pinpoint spray gun. Minimum intensity, 4; maximum intensity, 10. Each merged image was superposed with the IMS image on the measurement area image obtained 3, 24, and 72 h after the administration of the ADCs. At 24 h after the administration of the ADCs, the accumulation of MMAE released from human TF ADC was visibly higher than that of the control ADC. (**c**) The contents of MMAE in the tumour tissues 3, 24, and 72 h after the administration of the control ADC were 985 ± 500, 504 ± 222, and 92 ± 31 fmol/g (mean ± SD, *N* = 5 for each group), respectively. The contents of MMAE in the tumour tissues 3, 24, and 72 h after the administration of the human TF ADC were 785 ± 354, 2399 ± 637, and 353 ± 125 fmol/g (mean ± SD, *N* = 5 for each group), respectively. There was a significant difference between the contents of the control ADC and human TF ADC 24 h after administration (Tukey-Kramer, **P < 0.01, *N* = 5 for each group).

The signals originating from the MMAE released from ADCs were detected in the tumour tissues at 3, 24, and 72 h after the administration of the ADCs. The MMAE signal detected following the accumulation of the human TF ADC in the tumour tissue was greatest 24 h after administration, compared with the control ADC at the same time (Tukey-Kramer, *P* < 0.01, Fig. 4a,b). Tissue sections serial to those for MALDI-IMS were also quantified with LC-MS/MS (Fig. 4c), and the results were consistent with the IMS data from MMAE.

### High-resolution MALDI-IMS of MMAE in tumour tissues

**Figure 5:**
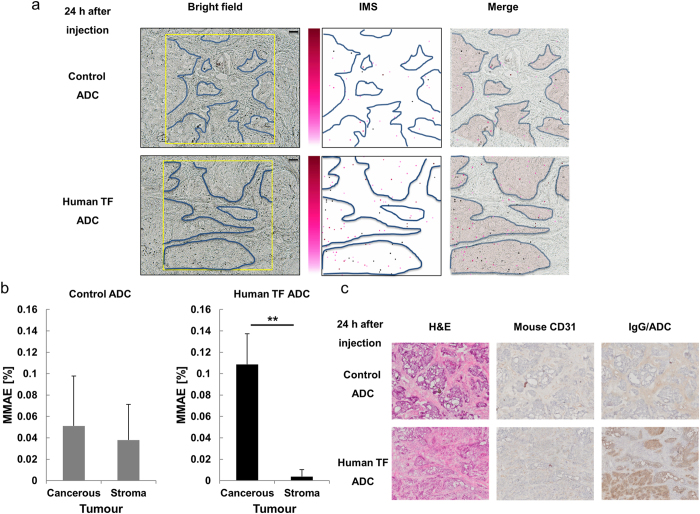
Distribution of the MMAE released from ADCs in tumour tissues. Original magnification, x10. The yellow rectangles on the bright field show the measurement area (1200 × 1200 μm). Pixel size, 10 μm; scale bar, 100 μm. The blue curved lines show the borders between the cancerous and stroma parts in the BxPC-3 tumour tissues. The MMAE images in the tumour tissues obtained from *m*/*z* 496.3 using MALDI-IMS are shown as pseudo-colour images (Red-Purple). The 5.0-mg/mL CHCA solution was applied with a pinpoint spray gun. Minimum intensity, 4; maximum intensity, 10. The merged images show the superposition of the IMS images on the measurement area image obtained 24 h after the administration of the ADCs. The red region of the merged images indicates the cancerous parts. (**a**) At 24 h after the administration of the human TF ADC, MMAE was distributed predominantly within the cancerous tissues. (**b**) The accumulation of MMAE within the cancerous parts was higher than that within the stroma parts in the BxPC-3 tumour tissues (Student’s *t*-test, **P = 0.003, *N* = 3 for each group). (**c**) Immunohistochemistry (mouse CD31 and IgG/ADC) revealed the distributions of the vascular endothelial cells and IgG on the corresponding ADCs, and H&E staining is shown in the left column.

To determine the characteristics of the MMAE distribution 24 h after the injection of the ADCs, MMAE was imaged at a pixel size of 10 μm. After 24 h, the released MMAE was present at a significantly higher concentration in the cancerous part after the injection of the human TF ADC, compared with the control ADC (Fig. 5a,b). The proportion of areas in the cancerous parts that contained MMAE after the injection of human TF ADC after 24 h was higher than that in the stroma parts (Student’s *t*-test, *P* = 0.003). Haematoxylin and eosin (H&E) staining revealed the histology of the BxPC-3 tumour tissue, which was composed of cancerous and stroma parts. Anti-mouse CD31 immunohistochemistry revealed the distribution of vascular endothelial cells in the BxPC-3 tumour tissue sections. CD31 was expressed in the stroma parts. Anti-rat IgG immunohistochemistry revealed that the anti-human TF mAb or its corresponding ADC was observed mainly in the cancerous parts. In contrast, the control mAb or its corresponding ADC was observed mainly in the stroma parts (Fig. 5c).

## Discussion

Nearly 50 individual ADCs are currently being examined in clinical trials, and 62% of the ACAs used for the ADCs are auristatin analogues, such as MMAE and monomethyl auristatin F (MMAF)^20^. Optimized linker design is required to release ACAs selectively at tumour sites. To improve the therapeutic window of an ADC, efforts have been made to improve linker technology over the past decade. We conducted quantitative and a semi-quantitative analyses of MMAE in BxPC-3 subcutaneous tumours by using LC-MS/MS and MALDI-IMS, respectively.

Unlike a pharmacological study using conventional tools, such as HPLC and LC/MS, MALDI-IMS can provide additional information about the distribution of MMAE in tumour tissues. Despite recent progress in MALDI techniques, it remains difficult to ionize macromolecular proteins^21^,^22^. The MALDI-IMS technique used in our study is specifically designed for low-molecular weight substances. Although we attempted to directly ionize the mAb and ADC in the tumour tissue by selective fragmentation with different proteases, it was difficult to determine MMAE alone, mAb, and ADC. However, our MALDI-IMS technique is valuable for optimizing the ADC. The present study clearly showed that MALDI-IMS can be used to visualize only free MMAE without interference from MMAE conjugated to mAb. Thus, MALDI-IMS is a novel evaluation method for the semi-quantitative determination of the intratumour distributions of MMAE released by ADCs.

The selection of a proper rational matrix is important to ionize MMAE using MALDI. We tested several matrixes, including 2, 5-dihydroxybenzoic acid, 9-aminoacridine, sinapine acid and CHCA, and found that CHCA was the optimal matrix for the IMS analysis of MMAE in tumour tissue. A CHCA solution containing 2.0-mM sodium acetate and aniline promoted the ionization of MMAE to create sodium adducts. According to the MS/MS peak in the present study–*m/z* 496.3, which was selected as an MMAE-specific fragment peak–MMAE can be observed with high reliability and accuracy in tumour tissues. MALDI-IMS was able to detect 1.0 fmol of MMAE, although the signal intensity was low. However, some aspects of the ionization uncertainty might not be captured by MALDI classification and are otherwise difficult to quantify. Further studies examining the adequacy of quantification using MALDI-IMS are needed.

The laser beam diameter used corresponded to a spatial resolution of 10 μm. This resolution allowed us to discriminate between the cancerous and cancer stroma parts of tumour tissue. However, cellular-level investigations remain impossible because the diameters of cells are typically 5 to 25 μm. Thus, improved resolution is needed to investigate whether MMAE is located within the tumour cells. However, MALDI-IMS represents a step forward, revealing the accumulation of MMAE released from human TF ADCs in the cancerous part. This distinctive accumulation of MMAE is reasonable given the high antitumour effects of human TF ADCs against the TF-positive BxPC-3 tumour.

In conclusion, MALDI-IMS is expected to be a powerful tool for design optimization of ADCs in the preclinical stage.

## Materials and Methods

### ADCs

Previously, we have developed several clones of anti-human TF mAb. Clone 1849 rat IgG2b has a high affinity for the TF antigen, whereas clone 372 rat IgG2b does not bind the TF antigen at all. Thus, we used clone 372 as an isotype-control mAb. The Val-Cit-MMAE consisted of a maleimide serving as the connection to the mAbs, PEG12 (Quanta BioDesign, CA, USA) to increase the polarity, a Val-Cit dipeptide to trigger cleavage by intracellular proteases, and a para-amino benzyl carbamate (PABC) as a self-immolative spacer for efficient MMAE release. MMAE (Medchem Express, NJ, USA) was dissolved in dimethyl sulfoxide (10 mM) and stored at −80 °C. The ADCs were produced by reducing the inter-chain disulfide bonds. The DAR of the ADCs was 2–4, as determined by the colourimetric measurement of thiol groups in biological samples using Ellman’s reagent, DTNB.

### Pancreatic cancer cell line BxPC-3 xenograft model

The human pancreatic cancer cell line BxPC-3 was obtained from the American Type Culture Collection (Manassas, VA). BxPC-3 cells were maintained at 37 °C in a humidified atmosphere with 5% CO_2_ and grown in RPMI-1640 medium (Wako, Osaka, Japan) containing 10% foetal bovine serum (FBS; Gibco, Grand Island, NY), 100-U/mL penicillin, 100-μg/mL streptomycin, and 0.25-μg/mL amphotericin B (Wako).

Six-week-old female BALB/c nude mice (CLEA Japan, Tokyo, Japan) were used. The anesthetized mice were subcutaneously inoculated with 1.0 × 10^6^ BxPC-3 cells suspended in 100 μL of phosphate-buffered saline (PBS) on the right back. The mice were injected with the agents (MMAE alone, human TF ADC, or control ADC) when the tumour volume (TV) reached 350 ± 150 mm^3^ (5 mice per group, *N* = 5). The TV was calculated using the following formula and the length (L) and width (W) of the subcutaneous tumour: TV = (L × V^2^)/2. The mice were maintained in cages under specific pathogen-free conditions, provided with standard food, and given free access to sterilized water.

To evaluate the distribution of MMAE in the tumour tissue, 0.2-mg/kg MMAE alone and 10-mg/kg ADCs were injected intravenously into the tail vein. The tumour tissue samples were surgically removed from a xenograft model at 3, 24, or 72 h after the administration of the agents. The samples were wrapped in gauze, frozen in dry ice powder, and stored in a −80 °C freezer until further analysis. For analysis, the samples were sectioned at a thickness of 10 μm and transferred to an indium tin oxide (ITO)-coated glass slide (Sigma, St. Louis, MO), and the tissue sections were dried before matrix coating. Continuous sections were stained with H&E (Muto Pure Chemicals, Tokyo, Japan) and immunohistochemistry.

The study was approved by the Committee for Animal Experimentation of the National Cancer Center, Tokyo, Japan. All animal procedures were performed in accordance with the Guidelines for the Care and Use of Experimental Animals established by the Committee. These guidelines meet the ethical standards required by law and also comply with the guidelines for the use of experimental animals in Japan.

### MALDI-IMS using mass microscopy

The IMS analysis was performed using an atmospheric pressure MALDI-ion trap (IT)-time-of-flight (TOF) mass spectrometer (prototype Mass Microscope; Shimadzu, Kyoto, Japan). The MALDI-IMS used in our study is specifically designed for detecting low-molecular weight analytes (with a mass detection range from *m/z* 50 to *m/z* 5,000).

For the application of MALDI to MMAE imaging, CHCA (Nacalai, Kyoto, Japan) in 75% acetonitrile, 0.02% trifluoroacetic acid, 2.0-mM sodium acetate and a 1/1000 dilution of aniline were used. To determine the definition of the MS and the MS/MS analysis of MMAE, 1.0 μL of a 10-mg/mL CHCA solution was used for the ionization of an MMAE standard (1.0 μL) onto an ITO slide. The mass spectrum was obtained by summing the signal intensities in the measurement areas. The CHCA on the tissue section was applied with a pinpoint spray gun (GSI Creos, Tokyo, Japan). Then, a 5.0-mg/mL CHCA solution was used for the ionization of the tissue samples. The total amount of CHCA per ITO slide was 30–35 mg.

MS and MS/MS analyses were performed in positive-ion mode within a mass range of *m/z* 150–770 for MMAE, with spatial resolutions of 10 and 20 μm. The laser was modulated to 80–160 shots/spectrum with a frequency of 400–800 Hz, and the laser power was set to 50–65 using the Mass Microscope operation software. The MS/MS analysis was performed with the collision-induced dissociation (CID) function of the quadruple ion trap cell on the Mass Microscope. The *m/z* 496.3 fragment ion was generated when [M + Na]^+^ was used as a precursor ion in the MS/MS analysis. The MMAE distribution mapping was performed using BioMap (Novartis, Basel, Switzerland) with the *m/z* 496.3 fragment ion signal obtained by MS/MS analysis. The non-treated BxPC-3 tumour tissues were used as a negative control to determine the baseline of the MS/MS analysis. The IMS data are shown as pseudo-colour images (Red-Purple). The baseline intensity had a value of 3; therefore, the minimum intensity was set to 4. To achieve a visible IMS signal, the maximum intensity was set to 10. The ratio [%] of the areas occupied by MMAE [μm^2^] to the whole measurement areas [μm^2^] in the tumour tissues was measured with ImageJ software^23^.

### LC-MS/MS

The samples were analysed using a liquid chromatograph tandem mass spectrometer API3200 LC-MS/MS system (AB SCIEX, Framingham, MA). The analytical column, a reversed-phase LC column (4.0 μm polar, 80 Å; 50 × 3.0 mm, Synergi; Shimazu), was heated to 40 °C. The injection volume was 10 μm, and the flow rate was 0.4 mL/min. The autosampler was equipped with a cooling stack set at 4 °C. Acetonitrile and a 0.1% (w/v) formic acid solution were used as the mobile phases. For the gradient elution, the mobile phase composition was as follows: 5% acetonitrile for 1.0 min, increased to 40% for 2.0 min, increased to 100% for 2.0 min, maintained at 100% for 2.0 min, and then decreased back to 5.0% for 2.0 min. The mobile phase was introduced into the spectrometer via electrospray ionization in positive-ion mode under multiple reaction monitoring (MRM) conditions. The MRM transitions were used for MMAE (*m/z* 718.4/152.2) and MMAF as an internal standard (*m/z* 732.4/170.3). The standard curve had a linear range from 0.10 nM to 100 nM. Samples from the non-treated BxPC-3 tumour tissues were used as a negative control.

### Immunohistochemistry

The 10-μm frozen sections were fixed with 4% paraformaldehyde (Wako) for 10 min. To block endogenous peroxidase, 3% hydrogen peroxide for 20 min was used. After being blocked with 3% skim milk in PBS for 30 min, the sections were incubated with an anti-rat IgG goat antibody (Histostar; MBL, Nagoya, Japan) for mAb/ADC according to the manufacturer’s instructions. The continuous sections were incubated with an anti-mouse CD31 goat antibody for vascular endothelial cells (10 μg/mL; R&D Systems, Minneapolis, MN) for 1 h at room temperature. After being washed with PBS, the sections were incubated with a horseradish peroxidase (HRP)-conjugated anti-goat IgG antibody (Jackson IR, West Grove, PA) according to the manufacturer’s instructions. Counterstaining was performed using haematoxylin. Negative controls included the replacement of the primary antibody by PBS and isotype antibodies.

### Statistics

Measured differences were considered significant at *P* < 0.05. For the quantification of MMAE using LC-MS/MS, statistical significance was determined using the Tukey-Kramer multiple comparison test. To distinguish between MMAE alone and MMAE conjugated to a mAb, statistical significance was determined using the Tukey-Kramer multiple comparison test. For the high-resolution MALDI-IMS of MMAE in tumour tissues, statistical significance was determined using the two-sided Student’s *t*-test. The bar graph data were expressed as the mean ± standard deviation (SD). The error bars indicated the SD. The statistical analyses were performed using Statcal QC (The publisher OMS, Saitama, Japan).

## Additional Information

**How to cite this article**: Fujiwara, Y. *et al.* Imaging mass spectrometry for the precise design of antibody-drug conjugates. *Sci. Rep.* **6**, 24954; doi: 10.1038/srep24954 (2016).

## Figures and Tables

**Table 1:** MS and MS/MS of MMAE.

MS	MMAE	
	*m/z*	
(a)		
[M + H]^+^	718.4	
[M + Na]^+^	740.4	
[M + K]^+^	756.4	
(b)		
Precursor ion	[M + H]^+^	321.2
		506.3
		687.4
	[M + Na]^+^	496.3
		682.3
		722.4
	[M + K]^+^	512.2
		544.3
		698.3

(a) The three positive-ion peaks derived from MMAE were observed by MS analysis: single-charge hydrogen and sodium and potassium adducts, denoted as [M + H]^+^, [M + Na]^+^, and [M + K]^+^, respectively. (b) MS/MS fragments of MMAE determined from each of the three positive-ion peaks of MMAE.
